# A cluster randomized trial of interferon ß-1a for the reduction of transmission of SARS-Cov-2: protocol for the Containing Coronavirus Disease 19 trial (ConCorD-19)

**DOI:** 10.1186/s12879-021-06519-4

**Published:** 2021-08-13

**Authors:** Carolina Iturriaga, Nat Eiffler, Rad Aniba, Rym Ben-Othman, Guillermo Perez-Mateluna, Jessica K. V. Meyer, Eleanor N. Fish, Tobias R. Kollmann, Nicolas Severino, Stephen Stick, Arturo Borzutzky, Cecilia Perret, José A. Castro-Rodriguez, Diego Garcia-Huidobro

**Affiliations:** 1 School of Medicine, Pontificia Universidad Católica de Chile, Vicuña Mackenna 4686, Macul, Santiago, Chile; 2 Telethon Kids Institute, Perth, Australia; 3 School of Medicine and Dentistry, University of Rochester, Rochester, NY USA; 4 Toronto & Department of Immunology, Toronto General Hospital Research Institute, University Health Network, University of Toronto, Toronto, Canada

**Keywords:** COVID, COVID-19, Coronavirus, Interferon, Protocol, Transmission, Prevention

## Abstract

**Background:**

SARS-CoV-2 infection rapidly spreads in populations due to the high rates of community transmission. Interrupting the shedding of SARS-CoV-2 may reduce the incidence of Coronavirus Disease 19 (COVID-19). Herein we provide a protocol for a cluster randomized trial that will examine the effectiveness of treatment with interferon (IFN) ß-1a compared to standard of care in limiting the transmission of SARS-CoV-2. Co-primary objectives are to determine whether IFN therapy reduces (a) the proportion of infected cases shedding SARS-CoV-2 at day 11 post randomization and (b) the incidence of transmission of SARS-CoV-2 infection from index cases to treatment-eligible household post-exposure contacts at day 11 after randomization. Secondary objectives include assessing the impact of IFN treatment on duration of viral clearance, hospitalizations and fatalities, and evaluating the safety of IFN treatment.

**Methods:**

Three hundred and ten households, each including an index case with a recent COVID-19 diagnosis and at least one asymptomatic treatment-eligible household contact, will be randomized to receive 3 doses of 125 μg IFN ß-1a by subcutaneous administration (days 1, 6, and 11), or standard of care. All participants will be followed until day 29.

**Discussion:**

The results from this trial will identify whether IFN ß treatment of mild or moderate COVID-19 cases accelerates viral clearance and prevents disease progression and whether IFN ß treatment of post-exposure contacts of COVID-19 cases reduces transmission of infection.

*Trial Registration:* This trial is registered at ClinicalTrials.gov NCT04552379; date of registration September 17, 2020.

## Background

Since the emergence of coronavirus disease 19 (COVID-19) in China in December 2019, there have been over 190 million cases worldwide and more than 4 million deaths to date [1]. Following the Severe Acute Respiratory Syndrome (SARS) in 2002 [2] and Middle East Respiratory Syndrome (MERS) in 2012 [3], SARS-CoV-2 has emerged as the most serious human coronavirus infection in 20 years. In approximately 80% of cases, SARS-CoV-2 infection results in mild to moderate COVID-19, with symptoms similar to common respiratory diseases such as influenza-like illnesses [3]. In 14% of cases, SARS-CoV-2 infection results in severe disease requiring oxygen supplementation and/or mechanical ventilation, with a further 2–75% progressing to respiratory failure, septic shock and/or organ failure [4, 5].

Even with the recent availability of vaccines, global herd-immunity remains elusive. Worldwide efforts to reduce SARS-CoV-2 infection rates continue, with preventative measures including distancing, use of facemasks in public places, and hand hygiene measures. Many countries have also implemented mandatory quarantine and contact tracing [6]. Individuals infected with SARS-CoV-2 shed virus and may be contagious for up to 5 days prior to developing symptoms (‘pre-symptomatic transmission’) [7–9]. Notably, nearly 60% of all infected subjects can shed virus pre-symptomatically [10]. Pre- or even asymptomatic shedding occurs across all age groups, even in children [11], and has contributed to the rapid expansion of the pandemic [7, 10]. Since pre-symptomatic shedding of virus can start up to 5 days prior to symptom onset, any post-exposure prophylaxis intervention for contacts recently exposed to a case could interrupt the spread of the virus.

With this strategy in mind, we have focused on the potential of post-exposure prophylaxis with interferon (IFN). Type I IFNs, IFN-αs/ß, are associated with the earliest innate immune response to viral infections. Type I IFNs invoke direct antiviral effects against different stages of viral replicative cycles and also activate immune cells to mediate viral clearance [10]. Certainly, the scientific literature is replete with evidence that pre-treatment of cells or small animals with an IFN-α or an IFN-β can protect from subsequent viral challenge. Type I IFNs were approved for therapeutic use in the context of chronic hepatitis C virus infection, in the 1990s. Notably, type I IFNs have seen limited clinical use for acute virus infections.

A clinical study that evaluated the therapeutic potential of a type I IFN against SARS, demonstrated rapid resolution of lung abnormalities in hospitalized cases treated with IFN-α [12]. More recently, results from an exploratory study undertaken in Wuhan, China, at the start of the SARS-CoV-2 pandemic, provided evidence that IFN treatment of moderate COVID-19 cases led to diminished lung pathology and accelerated viral clearance from the airways [13, 14]. Viewed altogether, the data suggest that IFN treatment for acute respiratory coronavirus infections may offer therapeutic benefits. Given the high SARS-CoV-2 transmission rates, especially with the emerging variants of concern, and the uncertainty surrounding achieving global herd immunity with vaccines, the major objective of this trial is to examine the safety and efficacy of post-exposure prophylaxis with IFN ß-1a in close contacts of confirmed COVID-19 cases. Herein we report the protocol of the Containing Coronavirus Disease 19 (ConCorD-19) study.

## Methods/Design

### Study overview

ConCord-19 is a prospective, cluster randomized trial of pegylated IFN ß-1a (PLEGRIDY®) treatment versus standard of care for index cases positive for SARS-CoV-2 infection and their household contacts. Index cases will be identified from acute respiratory COVID-19 clinics, primary care clinics and hospitals, reporting evidence of SARS-CoV-2 infection by RT- PCR. Household contacts will be identified via the index cases and will be approached to participate following consent from the index case.

**Fig. 1:**
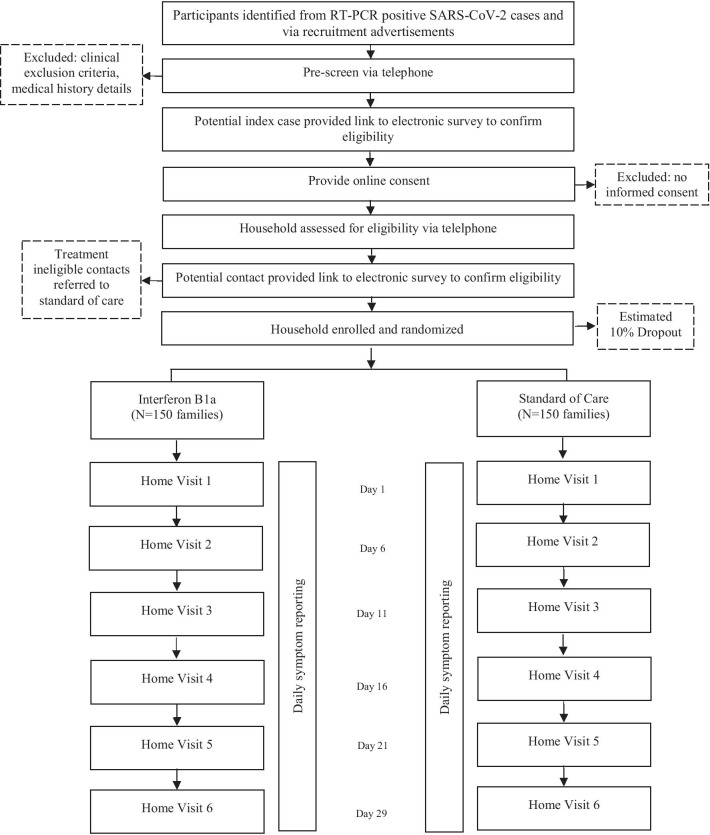
Diagram of ConCorD-19 study

Households will be randomized to receive IFN ß-1a or clinical care recommended by the Ministry of Health or the patient´s medical provider. Study participants will continue to follow national guidelines regarding self-isolation and infection prevention during study participation. The study design is described in Fig. 1.

### Primary and secondary objectives

The two co-primary objectives of this study are:

1. To determine whether IFN ß-1a treatment reduces the proportion of infected cases shedding SARS-CoV-2 at day 11 after randomization.
2. To determine whether IFN ß-1a treatment reduces the incidence of SARS-CoV-2 transmission from index cases to treatment-eligible household contacts, as measured by any positive upper airway infection evidenced by real-time Polymerase Chain Reaction (RT-PCR), in contacts at day 11 after randomization.

Secondary objectives of this study are:

1. To determine whether in index cases infected with SARS-CoV-2, IFN ß-1a treatment compared with standard of care reduces the duration of SARS-CoV-2 upper airway virus shedding over 28 days following randomization.
2. To determine whether IFN ß-1a treatment affects the incidence of SARS-CoV-2 upper airway infection or serological conversion by study day 29 in household contacts.
3. To determine whether IFN ß-1a treatment compared with standard of care reduces the proportion of infected cases that require hospital admission or die due to COVID-19.
4. To determine the safety of IFN ß-1a in the treatment and prevention of SARS-CoV-2 infection.

### Setting

The study will take place in the Santiago metropolitan area in Chile, population 7,112,808 [15]. Chile’s healthcare system has private and public providers, and insurance systems. Country-wide, Chileans have access to good primary health care and hospital networks, achieving a life expectancy that places Chileans in the global upper quintile [16]. In Chile, the first case of COVID-19 was confirmed on March 3, 2020, [17] with 608,973 confirmed cases and 16,608 confirmed deaths in 2020 [18].

### Study population and inclusion/exclusion criteria

**Fig. 2:**
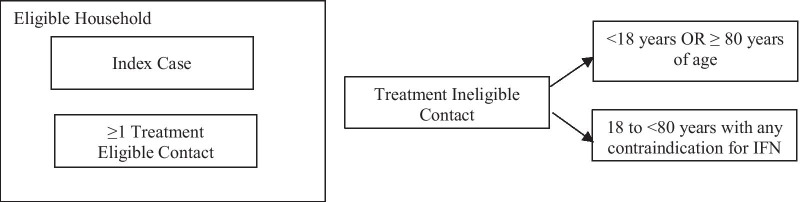
Household and participant eligibility

The study population will include individuals, between the ages 18 and < 80 years, who have tested positive for SARS-CoV-2 (RT-PCR) within the previous 72 h (Index Cases) and their household contacts (Eligible Contacts, between ages 18 and < 80 years, and Ineligible Contacts). Eligible households must include at least one eligible index case and one eligible household contact. Treatment ineligible participants will also be enrolled, to assess SARS-CoV-2 transmission within a household. Details of household and participant eligibility are proved in Fig. 2 and Table 1.

**Table 1:** Participant inclusion and exclusion criteria

	Inclusion criteria	Exclusion criteria*
Index cases	• Provided a signed and dated informed consent form • Aged 18 to < 80 years of age • Confirmed SARS-CoV-2 diagnosis by PCR within 72 h of first treatment dose • The first known diagnosis in the household • Not currently enrolled in a clinical trial and agree to not take any other investigational treatments over the next 28 days • Must plan to remain resident in the household during the study • Lives in household with at least one other ‘treatment-eligible household contact	• Inability to take medications orally or by injection • Known sensitivity/allergy to interferons or use of interferons for another indication • Known adverse drug-drug interactions with any study drugs • Malignancy • Known clinical immune deficiency • Pregnancy or unwillingness of female participant of childbearing age to use recognized methods of birth control/contraception during the trial period • Retinopathy • Known grade 4 or 5 chronic kidney or liver disease • Known arrhythmias • Known autoimmune disease or chronic inflammatory disease • Chronic liver disease • Hospitalization for depression in the last 3 months • Current suicidal ideation • Previous therapeutic use of IFN
Treatment eligible contact	• Provided a signed and dated informed consent form • Aged 18 to < 80 years of age • Not currently enrolled in a clinical trial and agree to not take any other investigational treatments over the next 28 days • Must plan to remain resident in the household during the study • No history of previously confirmed SARS-CoV-2 diagnosis	
Treatment ineligible contact	• Provided a signed and dated informed consent form, parental informed consent, and assent if applicable • Under 18 years OR ≥ 80 years of age OR aged 18 to < 80 years with any contraindication for IFN treatment listed in ‘exclusion criteria’ • Not currently enrolled in a clinical trial and agree to not take any other investigational treatments over the next 28 days • Must plan to remain resident in the household during the study • No history of previously confirmed SARS-CoV-2 diagnosis	

^*^All household members are ineligible if participation by any member is declined or if the index case has been in complete self-quarantine from other household members during the 48 h prior to diagnosis of SARS-CoV-2 infection

### Recruitment

Participants will be identified from the list of RT-PCR positive SARS-CoV-2 cases obtained daily from the UC Christus Health Network virology laboratory, COVID-19 outpatient clinics, or the Emergency Rooms. This is a large private, academic health network in Santiago, Chile. Additional patients will be recruited from City Health Departments, local primary care clinics, and through advertising in the community. The recruitment period will continue until the enrollment target is reached.

Cases will be contacted by telephone to explain the study, invite participation, and confirm eligibility. A member of the research team will contact the index case via telephone for a pre-screening discussion (clinical inclusion and exclusion criteria, medical history details and verbal consent). If the participant is eligible to participate, he/she will be provided with a link to an electronic survey, to confirm eligibility and to provide consent to participate. Once an index case has indicated willingness to participate in the study, household contacts will be assessed for eligibility. If at least one household member is considered eligible, he/she will be sent a link to an electronic survey to confirm the reported information. During the recruitment telephone calls, treatment ineligible contacts in each household will be identified and invited to participate in the study.

This pre-consent online approach will reduce face-to-face contact time and the possible exposure of study staff and participants to infection by ensuring all participants are consented and eligibility confirmed, prior to Study Day 1.

### Baseline and follow-up home visits

Refer to Table 2. To maintain isolation of index cases and their household contacts, all biological samples for the study will be collected during home visits. Home visits will be conducted by appropriately trained trial staff. Staff teams will be composed of a physician and a nurse. All will be trained in Good Clinical Practice and study procedures.

**Table 2:** Protocol for home-visit procedures and data collection

	Procedures
Day 1	Medical history Brief physical examination Vital signs (forehead temperature, pulse, blood pressure, pulse oximetry) Document weight and height Review baseline symptoms, if applicable Document concomitant medications and therapies Baseline adverse event assessment Saliva sample collection Nasal brushing for transcriptomics Nasal lining fluid sampling for IgA Blood sample collection (2 tbsp; about 30 ml) Dispense and instruct on use of diary cards Dispense individual thermometers for each participant Urine sample collection and pregnancy test pack, if applicable Administer acetaminophen 1000 mg PO pre-medication, if applicable Study drug administration, if applicable Observation for 20 min post study drug administration, if applicable General education to patients and family members about COVID-19 standard care
Day 6	Brief physical examination Vital signs (forehead temperature, pulse, blood pressure, pulse oximetry) prior to study drug administration Review adverse events Review concomitant medications Saliva sample collection (immediately prior to study drug administration) Nasal lining fluid sampling for IgA Blood sample collection (2 tbsp; about 30 mL) Urine sample collection and pregnancy test pack, if applicable Administer acetaminophen 1000 mg PO pre-medication, if applicable Study drug administration, if applicable Collect completed diary cards and dispense new diary cards Observation for 20 min post study drug administration, if applicable
Day 11	Brief physical examination Vital signs (forehead temperature, pulse, blood pressure, pulse oximetry) prior to study drug Review adverse events Review concomitant medications Collect completed diary cards and dispense new diary cards Saliva sample collection (immediately prior to study drug administration) Nasal lining fluid sampling for IgA Blood sample collection (2 tbsp; about 30 ml) Urine sample collection and pregnancy test pack, if applicable Administer acetaminophen 1000 mg PO pre-medication, if applicable Study drug administration, if applicable 12) Observation for 20 min post study drug administration, if applicable
Day 16 & 21 (± 24 h)	Diary completion Saliva sample collection
Day 29 (± 24 h)	Brief physical examination Vital signs (forehead temperature, pulse, blood pressure, pulse oximetry) Review adverse events Review concomitant medications Collect completed diary cards Blood sample collection Saliva sample collection Closure of study

Day 1 procedures must be executed within 72 h of a confirmed COVID-19 index case (RT-PCR evidence of SARS-CoV-2). During the first visit, participants will read and sign an informed consent form available as online appendices. Each household will also be randomized to IFN ß-1a treatment or standard of care. All study participants, regardless of their assignment, will be asked to monitor any symptoms of illness during the trial and, in particular, any ‘flu-like’ symptoms for the first 4–24 h following IFN ß-1a treatment, using an electronic diary. Study participants will be instructed to follow all recommendations from the Ministry of Health and advice from their medical providers in regard to COVID-19 clinical care. If participants are hospitalized during the study, all clinically relevant treatments will be provided as per health guidelines. Consent will be requested for access to medical records relating to any hospitalization or clinic visit.

Participants in the IFN ß-1a treatment arm will be alerted to potential influenza-like symptoms that are sometimes associated with IFN treatment and will be advised to take the provided acetaminophen (1000 mg 4 times per day) from the time of IFN administration until 24 h post-injection, to reduce the likelihood of these symptoms. Participants in the control arm will also be instructed to take the provided acetaminophen within 24 h of the first home visit, to conform with the intervention group. Participants will receive information on the potential risks of IFN ß-1a treatment during pregnancy and other contraindications for PLEGRIDY® use.

### Biospecimen collection, processing, and storage

Peripheral blood, nasal samples and/or saliva will be collected from index cases and eligible household contact members at each study visit. Refer to Table 2. Samples will be taken by experienced staff, trained in the home visit procedures. All samples will be kept on ice and transported to the laboratory.

Each saliva sample will be collected by passive drool collection and spitting into a sterile container. Saliva samples intended for viral PCR will be collected in phosphate saline buffer (PBS) and kept on ice until final storage in the laboratory. Participants will be asked not to eat, smoke, drink or brush their teeth 30 min prior to the collection. Saliva collection will be followed by collection of nasal fluid samples using a synthetic absorptive device (Nasosorption FX, NSFL-FX-11 or NSFL-FXI-13), that absorbs the mucosal lining fluid. A second nasal sampling will consist of inserting an interdental brush into the nostril and gently twirling the brush against the inferior turbinate which runs along the lateral wall of the nasal passage. After collection, the brush is inserted into a sterile tube pre-filled with RNAlater (Life technologies cat# AM7021) to allow RNA stabilization until analysis. Blood samples are collected last, and a total of 23 ml of peripheral blood will be obtained from an easily accessible vein by experienced staff. A 21G butterfly needle will be used to draw blood into 3 distinct collection tubes; IVD PAXgene blood collection tube (BD, Cat. No. 762165) for RNA samples, CTP Vacutainer (BD, Cat. #362761) for mononuclear cells (PBMC) collection and Lithium Heparin vacutainers (BD, cat# 367526) for the remainder. At the end of collection, all biospecimens will be transported to the laboratory for further processing and/or storage.

Saliva, nasal lining fluid samples and nasal brush collection tube contents will be aliquoted in cryovials and stored at -80 °C. Blood PAXgene tubes will be kept at room temperature for a minimum of 2 h for complete lysis of blood cells and then stored at − 80 °C for subsequent transcriptomic analysis. 200 µl of heparinized blood will be transferred to a cryovial and stored at − 80 °C without any further processing, for subsequent epigenetic analysis. A total of 600 µL of whole blood will be processed as previously described [19] using Smart tube lysis and fixing solutions as per the manufacture’s recommendations and stored in cryovials at − 80 °C for subsequent flow cytometric analysis. Plasma will be separated from the remainder of the blood by centrifugation and aliquote for storage at − 80 °C. All specimen aliquots will be barcoded and sample location as well a specific processing information recorded electronically, to ensure optimal tracking and standardization.

### Treatment assignment and randomization

Households will be randomized as clusters to receive either IFN ß-1a treatment or standard of care, once a household meets all the inclusion and none of the exclusion criteria. Households will be randomized to IFN ß-1a treatment or standard of care at a 1:1 ratio. The minimization technique (biased coin, *p* = 0.7) will be applied to achieve balance between groups stratified by the total number of people within the household. This will be implemented through a platform provided by the Telethon Kids Institute (https://qminim.telethonkids.org.au/).

Households will be randomized during the first home visit, once eligibility is confirmed, participants have signed informed consent, and baseline data collection procedures are completed. Field team members will contact the research laboratory, at which time the household assignment to treatment or standard of care will be made by the randomization software. Participants and study staff will not be blinded to randomization of treatment or standard of care, but all data analyses will be blinded to allocation.

### Intervention arm

Participants (index cases and treatment eligible household contacts) will receive 125 µg of pegylated IFNß-1a (PLEGRIDY®, Biogen) administered on Study Days 1, 6 and 11 (i.e. for a total of 3 doses). IFN ß-1a will be administered at the home of the participant, via subcutaneous injection from appropriately trained trial staff, for whom administration of subcutaneous injections is within their normal scope of practice (e.g. nurse or physician). The staff will remain at the home for 20 min after the last individual in a household receives their dose of IFN ß-1a, before leaving the residence, to ensure no immediate adverse events associated with IFN administration occur. All field team staff will be supplied with appropriate personal protection equipment, trained in the use of this equipment and in protocols for infection control, and will always attend in pairs. Staff administration of IFN ß-1a will ensure medication adherence.

### Control arm

Participants randomized to the control group will receive standard of care, including symptom-based supportive treatment provided by the individual’s personal medical provider. This could include acetaminophen, non-steroidal anti-inflammatory drugs, cough suppressants, antibiotics, inhaled bronchodilators. Participants in this arm will not receive any IFN ß-1a treatment or a placebo. This standard of care approach was selected as the comparator, since no other approved intervention medications are available for non-hospitalized cases.

All participants, regardless of their allocation group, will receive a brochure with the recommendations of the Chilean Government for patients with COVID-19, including household isolation. Within each household, the index case will be asked to isolate from their contacts.

### Data management and confidentiality

Clinical and laboratory data for this trial will be collected and managed using REDCap (Research Electronic Data Capture) electronic data capture tools hosted at the Telethon Kids Institute, Perth, Australia [20, 21]. REDCap is a secure, web-based application designed to support data capture for research studies, providing: (1) an intuitive interface for validated data entry; (2) audit trails for tracking data manipulation and export procedures; (3) automated export procedures for seamless data downloads to common statistical packages; and (4) procedures for importing data from external sources. Data will be stored securely on a Telethon Kids Institute server (restricted access), in accordance with the requirements of good clinical practice and Health Privacy Principles [18]. All paper files will be stored securely at the research lab. Only members of the research team will have access to data, including the final trial dataset. There are no contractual agreements that limit the investigators access to data. The dataset will be available to the public upon request and approval by the investigators.

### Data and safety monitoring

An independent Data and Safety Monitoring Board (DSMB) will be convened and will meet weekly for the first four weeks of the study and then monthly until database lock. The DSMB will monitor data completeness, duplication, rates of enrollment and participation, and the general study conduct. The DSMB will be composed of individuals with the appropriate expertise, including at least three independent clinicians and/or biostatisticians who, collectively, have experience in the management of biostatistics and the conduct and monitoring of randomized controlled trials. Members of the DSMB will be independent of trial conduct. The DSMB will review data from the treatment and control groups of the trial. At each meeting, a descriptive summary of withdrawals, serious adverse events and non-serious adverse events thought to be related to the study drug will be provided, along with outcome data for the primary outcomes. The DSMB will provide its input to the trial chief investigator.

Adverse events (AE) and adverse reactions (non-serious and serious) will be captured from the time of randomization until study day 29. For the purposes of this study, the investigator or delegate is responsible for recording all AE, regardless of their relationship to study treatment, with the following exceptions: (1) conditions that are pre-existing at enrollment and do not deteriorate; (2) abnormal laboratory values unless deemed clinically significant by the investigator and documented as such; (3) the onset of COVID-19 symptoms among asymptomatic household contacts or new symptoms within the household index case. The seriousness of an AE will be assessed by an investigator with the following exceptions: (1) elective surgery planned at the time of enrollment, and (2) hospitalization due to progression of disease will not be considered a serious adverse event (SAE) for the purposes of this trial.

All adverse events/serious adverse events will have their relationship to the trial intervention and severity assessed by the investigator who evaluates the adverse event, based on temporal relationship and his/her clinical judgment. All severe adverse events will be reported immediately to the sponsor, except for those SAEs that are identified as not requiring immediate reporting. The immediate reports should be followed promptly by detailed, written reports. Clinic trial liability insurance will cover costs for participants who suffer harm during trial participation. Investigators will ensure harmed participants receive treatment for their condition.

IFN treatment is known to induce injection site erythema and, in up to 80% of recipients, an ‘influenza like’ syndrome, comprising fever, headache and body aches. Importantly, there are no respiratory signs or symptoms associated with IFN treatment, i.e. the response to IFN treatment is different than COVID-19 disease. However, ‘flu-like symptoms’ also occur in COVID-19 disease, and could lead to an increase in clinical tests in the IFN treated cohort if not appropriately managed. There are several strategies that may mitigate this potential response to IFN treatment that already constitute standard practice for multiple sclerosis (MS) patients on IFN therapy. Participants will be informed about the likely side effects of IFN treatment and asked to monitor and manage these. Monitoring consists of self-assessment of symptoms (there are no respiratory symptoms or anosmia known to be associated with IFN use) and the time frame during which this ‘influenza like’ syndrome following IFN treatment occurs (restricted to the first 4–24 h after administration of an IFN dose). Management consists of advising the participant to take acetaminnophen (provided to the participant) from the time of IFN administration up to 24 h after, to reduce the likelihood of this ‘influenza like’ syndrome. This approach is recommended for individuals with MS receiving IFN therapy (see PLEGRIDY package insert product monograph) and reduces syndrome symptoms by 80%.

Interferon ß-1a is safe when taken during pregnancy, with no evidence of increased rates of congenital anomalies or spontaneous abortions, according to data reported by the European IFNbeta Pregnancy Registry. If a female subject is diagnosed as pregnant by a positive.

pregnancy test or other method of diagnosis, the principal investigator will notify the DSMB and Ethics Committee of this. If the subject is enrolled in the IFN arm and still has outstanding IFN doses to be administered, the decision whether to continue IFN treatment or not will be made by weighing the risk/benefit on a case by case basis.

### Treatment discontinuation, withdrawals, and loss to follow up

Participants may withdraw from the study at any time. Withdrawing from the trial will not affect their access to standard medical care. The treating medical team may also consider that continued participation is not in the best interest of the participant. In the case of discontinuation, the reason(s) for withdrawal will be documented. Consent to use study data, including data collected until the time of discontinuation and data to inform primary and secondary outcomes, will be requested specifically from participants. Following discontinuation, participants will receive standard care. Participants who withdraw will not be replaced.

### Protocol modifications

Protocol modifications will be decided by consensus among members of the research investigator team to ensure successful completion of the trial and to minimize compromising the integrity of the trial. Changes will be reported to the Institutional Review Board, DSMB, and the Chilean Public Health Institute, who oversee the conduct of the trial. The full protocol will be available to the public upon request and approval by the investigators.

### Data analyses

Analyses of data for primary and secondary objectives will be conducted per protocol and through intent-to-treat using the procedures detailed below and described in greater detail in a statistical analysis plan, available upon request. Data analysts will be blind to treatment allocation. Analyses will be conducted using R and statistical coding will be available to the public upon request and approval by the investigators.

#### Analysis for primary objective 1

The Primary Objective 1 is to determine whether IFNß-1a treatment reduces the number of COVID-19 cases still shedding SARS-COV-2 in saliva at day 11 following randomization. Statistical analysis of SARS-CoV-2 RT-PCR results from the index case population will be undertaken comparing IFN ß-1a treated index cases to index cases receiving only standard of care. A linear regression model (binomial family, logit link for dichotomous outcomes) will be applied to data collected, where a risk ratio (and 95% confidence interval) will be reported for the treatment arm variable, adjusted for age, sex, baseline viral load and vaccination status at enrollment.

#### Analysis for primary objective 2

The Primary Objective 2 is to determine whether IFN ß-1a treatment reduces the incidence of SARS-CoV-2 transmission from an index case to treatment-eligible household contacts, as measured by upper airway RT-PCR in contacts at day 11 after randomization. Statistical analysis of SARS-CoV-2 RT-PCR results from household contacts will be undertaken comparing households assigned to IFN ß-1a treatment or standard of care. A linear regression model (binomial family, logit link for dichotomous outcomes) with clustering within a household will be applied, where a risk ratio (and 95% confidence interval) will be reported for the treatment arm variable, adjusted for age, sex, baseline viral load of the index case, vaccination status of the index case, vaccination status of eligible contacts, contacts between index case and household contacts during the initial 11-day period, baseline RT-PCR and antibody levels of treatment eligible contacts at enrollment, number of household members and physical details of the household size, e.g. number of bathrooms, number of bedrooms.

#### Secondary analyses

Additional analysis of both primary objectives will be conducted to include more rigorous group comparisons, e.g. Fisher’s exact test of proportions and a stratified Cochran-Mantel–Haenszel test of proportions. If the primary analysis method, the generalized linear model with binomial family and log link, does not fit the data well, or converge, a logit link will be used, meaning an adjusted odds ratio and 95% confidence interval will be reported. Specifically, a regression model will be used where the outcome variable is Yes/No for SARS-CoV-2 from the day 11 saliva specimen, with odds ratio and 95% confidence interval reported treatment (active/control) variable, following covariate adjustments. Primary objective 2 analysis will include clustering within households, where appropriate. Secondary outcomes will be reported and analyzed as above for the primary outcomes. Poisson and/or ordinal regression analysis will be conducted to compare days of viral shedding between the IFN treated and standard of care groups. The Cox proportional hazards regression model, with similar adjustments for variables as above, will be employed to examine time based, and specifically time-censored, outcome variables between groups. In addition, Bayesian analyses will be estimated using data-generated distributions of viral shedding at days 11, 16, and 29, and household infection rates. These prior distributions will be used to generate posterior probabilities for the intervention arm.

#### Management of missing data

Information on why individuals chose not to participate or are lost to follow up will be collected. We anticipate few cases of missing data, assuming high adherence among participants and a low drop-out rate. However, to comply with the intent-to-treat analysis, all participants will be included in the data analysis. Hence, we will manage missing data using several strategies. First, if recorded data reports a change of outcome (e.g., change from positive to negative of a SARS-CoV-2 RT-PCR), we would carry forward that finding, as it is very unlikely that the result would change back to positive. Missing data where change has not occurred will be estimated using multiple imputation.

### Sample size and power considerations

Three hundred and ten households will be randomized to treatment or standard of care. Households will be randomized as clusters, assuming 4 individuals per household; approximately 1240 individuals will be enrolled. Data from Wuhan, China, suggest the proportion of untreated index cases still shedding virus at day 11 is ~ 85% [22]. A sample of 278 households (310 considering a 10% drop out rate) has > 90% power, at alpha 0.025, to detect a difference between the proportion of index cases shedding virus at day 11 between the treatment arm and standard of care arm, if the proportion is 65% in the treatment arm; based on a two-tailed Fisher’s exact test. In addition, the sample, including 834 household contacts (417 per arm), has > 90% power, at alpha 0.025, to detect an odds ratio of 0.5 for a reduction in transmission to a household contact; based on a two-tailed stratified Cochran-Mantel–Haenszel test, with intra-class correlation 0.15.

### Publications

Trial results will be communicated through manuscript publications, conference presentations, seminars, and through social media.

### Trial status

Participant recruitment started on December of 2020. At the time of manuscript submission, 251 households have been enrolled.

## Discussion

ConCorD-19 will identify whether early treatment with IFN ß1a offers a therapeutic advantage of accelerated resolution of COVID-19 and whether IFN ß1a treatment of post-exposure contacts protects from infection, thereby reducing transmission. The results from this trial may provide evidence and guidelines for a new outpatient treatment for COVID-19 with mild or moderate disease severity. Treatment interventions are needed, especially with the emergence of variants of concern that are highly transmissible, that may reduce the effectiveness of vaccinations [23].

## Data Availability

Not applicable.

## Abbreviations

AE: Adverse events
COVID-19: Coronavirus Disease 19
DSMB: Data and Safety Monitoring Board
IFN: Interferon
MERS: Middle East respiratory Syndrome
MS: Multiple sclerosis
RT-PCR: Real-time Polymerase Chain Reaction
REDCap: Research Electronic Data Capture
SAE: Serious adverse event
SARS: Severe Acute Respiratory Syndrome
